# Genetic evolution, phylodynamics, geographic spread of H9N2 avian influenza viruses in China from 2014 to 2025: an increasing potential zoonotic risk

**DOI:** 10.1080/22221751.2026.2713320

**Published:** 2026-08-03

**Authors:** Zifeng Pang, Peiting Zhong, Cuishan Mai, Junkun Fan, Yejia Wu, Yao Zhang, Hanlin Liu, Huapeng Sun, Ming Liao, Hailiang Sun

**Affiliations:** a College of Veterinary Medicine, South China Agricultural University, Guangzhou, People’s Republic of China; b Key Laboratory of Zoonosis Control and Prevention of Guangdong Province, South China Agricultural University, Guangzhou, People’s Republic of China; c National and Regional Joint Engineering Laboratory for Medicament of Zoonosis Prevention and Control, South China Agricultural University, Guangzhou, People’s Republic of China

**Keywords:** H9N2 influenza virus, genotype, geographic dispersal, host transition, pathogenicity

## Abstract

Subtype H9N2 of the avian influenza virus (AIV) poses a growing threat to the poultry industry and public health. However, since 2014, there has been no systematic study of its genetic evolution, genotype, spatial dynamics, and pathogenicity in China. Therefore, we performed a large-scale sequence analysis of the genome of Chinese H9N2 viruses from 2014 to 2025 using public databases and laboratory isolates. We identified 52 different genotypes in 1,591 H9N2 viruses, including 4 previously recognized genotypes (G6, G57, G58, and G68) and 48 newly defined genotypes (G118–G163) in this study. G57 and G118 were the main epidemic genotypes in China from 2014 to 2025. Bayesian phylogeographic analysis showed that there were 12 obvious migration paths for the spread of H9N2 AIVs in China from 2017 to 2022. In particular, the South China region was the main transmission centre. H9N2 AIV continues to circulate in chickens and ducks in China and spreads to other hosts. The H9N2 AIVs with the G57 and G118 genotypes could effectively replicate in MDCK, CEF, A549, and HBE cells with titres of 0.97–8.5 lgTCID_50_/mL. The G57 and G118 genotypes H9N2 viruses preferentially bound to α-2,6-linked sialic acid glycopolymers (human receptors), and effectively replicate in multiple organs of mice and chickens and cause pathological changes in the lungs. Thus, it is necessary to strengthen the monitoring and prevention of H9N2 AIVs in China.

## Introduction

The H9N2 subtype of avian influenza viruses (AIVs) has spread widely and is even chronically prevalent in poultry in Asia, Africa, and the Middle East. The subtype was first isolated from turkeys in 1966 [1] and is causing global concern. The host spectrum includes quails, chickens, ostriches, ducks, geese, pigeons, wild waterfowl, and terrestrial birds [2–4]. H9N2 AIVs can break through interspecies barriers and infect mammals, and infections have been reported in pigs in several provinces in China [5]. From 1998 to June 2021, a total of 87 human infections were reported [6, 7].

A serological survey of poultry-related workers showed that the rate of human H9N2 infection is much higher than expected, possibly due to infected patients usually showing only mild influenza-like symptoms. As a result, many human cases of H9N2 infection may go undetected [8, 9]. In addition, H9N2 AIVs serve as internal gene donors for other AIVs, such as H5N1, H7N9, H10N8, H5N6, H10N3, and H3N8, which enhances their adaptability to mammalian hosts [10–14]. A novel H7N9 AIV with six internal genes from the H9N2 virus has infected humans in China since 2013. In November of the same year, another new recombinant AIV, H10N8, was isolated from a 73-year-old woman, and its six internal genes were all closely related to the H9N2 virus circulating in Chinese poultry [15, 16]. H9N2 AIVs cause economic loss in the poultry industry and pose a potential threat to public health.

Different viral lineages and genotypes have been established through extensive and complex gene reassortment and mutation. Based on genetic evolutionary analysis of haemagglutinin (HA) genes, H9N2 AIVs can be divided into Eurasian and American lineages [17]. Most of the H9N2 strains circulating in China belong to Eurasian lineage, including A/chicken/Beijing/1/94-like (BJ-94-like) [18]. The H9N2 virus genes isolated in China from 1994 to 2013 have undergone extensive mutations, and the HA gene has formed 16 different branches. Based on whole-genome analysis, 117 genotypes have been identified, of which the G57 genotype has become the predominating genotype in China since 2013.

The six internal genes of the H7N9 influenza virus in 2013 were provided by the G57 genotype [19]. A study based on Bayesian analyses of the haemagglutinin (HA) gene has revealed that Clade 16 appeared for the first time in China in April 2012 and showed low cross-reactivity with Clade 15 viruses [20]. These data indicate that avian H9N2 viruses pose a potential threat to public health through direct infection or reassortment to generate new subtypes. Current evidence shows that the replication ability of H9N2 is significantly enhanced in human respiratory cells through continuous gene mutation and reassortment. Furthermore, its adaptability and transmission efficiency in mammalian models are improved, and its environmental stability is increased [21]. This leads to an increasing risk of cross-species transmission and human infections. However, there has been a dearth of systematic analyses of its genetic evolution, long-term genotype, geographical spread, and interspecies transmission in China since 2014.

We conducted a large-scale whole-genome sequence analysis of 1,583 strains isolated from China (retrieved from the GISAID and NCBI Influenza databases) and 8 H9N2 AIVs isolated in our laboratory in 2014–2025. We identified the genotype diversity and used Bayesian phylogeography to track the geographic migration and host transmission of H9N2 AIVs in different regions of China. We also investigated the replication ability and receptor binding affinity of viruses with the G57 and G118 genotypes in vitro, as well as their pathogenicity in chickens and mice.

## Materials and methods

### Ethics statement and biosafety

This study was carried out in ABSL-2 facilities in compliance with protocols approved by the biosafety committee of South China Agricultural University (SCAUADL2018-010). All animals involved in experiments were reviewed and approved by the Institution Animal Care and Use Committee at SCAU and were treated in accordance with the guidelines (SCAUADL2018-010; 12 October, 2017).

### Virus isolation and sequence preparation

Influenza surveillance of chickens was conducted from 2020 to 2022 in the provinces of Guangdong and Shandong in China, and H9N2 AIVs were isolated and purified. The total RNA was extracted according to the instructions of the RNAfast200 purification kit (Fastagen Biotech, Shanghai, China). RT-PCR and PCR were conducted using Uni12 and universal primers for influenza viruses [22]. The PCR products were recovered and cloned into the pMD-19 T vector (Takara Bio Inc., Beijing, China) for sequencing. Sequencing data were spliced using the Seqman program Lasergene (Version 7.1). The full-genome sequences of the viruses isolated in this study were deposited in the Global Initiative of Sharing All Influenza Data (GISAID) database.

All available genome sequences of H9N2 AIVs from 2014 to 2025 were retrieved from the GISAID and GenBank Flu databases, and the resulting sequences were classified using CD-HIT v4.6. The viral sequences were removed if they (a) did not have eight full segments, (b) had evidence of lab errors, or (c) had sequences with 100% similarity. Maximum-likelihood (ML) phylogeny sequence alignments were conducted using MAFFT version 7.149 [23]. An ML tree was constructed using PhyloSuite software (version 1.2.2) with the ultrafast bootstrap model, 10,000 bootstraps, and each gene classified according to an evolutionary lineage [24]. We used ITOL version 5 to complete the annotation of the evolutionary tree and to adjust it aesthetically [25].

### Phylogeographic inference

We conducted subsampling to reduce sample bias when obtaining the final dataset for our detailed phylogeographic analysis. A Bayesian stochastic search variable selection (BSSVS) model with asymmetric substitution was also used. For each independent dataset, multiple runs of the MCMC method were combined using LogCombiner (version 1.8.3) with 200,000,000 total steps for each set and sampling every 10,000 steps. Next, we used spreaD3 (version 0.9.6) to develop interactive visualizations of the dispersal process over time and to perform a Bayes factor (BF) test to assess the support for significant individual transitions between distinct geographic locations. Host-transfer analysis was performed using the same method [26, 27]. Only strains collected between 2017 and 2022 were used, as this period provided sufficient high-quality sequences with complete geographic and host metadata to ensure robust and reliable phylodynamic inference. A stratified sampling strategy based on region, host, and year was used to select strains to ensure representative coverage and minimize sampling bias, which was suitable for Bayesian spatial and host transmission analyses.

### Bayesian maximum cluster credibility phylogeny and evolutionary dynamics analysis

Multiple runs of the MCMC method were combined using LogCombiner (version 1.8.3) with 200,000,000 total steps for each set and sampling every 10,000 steps. Convergence of relevant parameters (i.e. effective sample sizes >200) was assessed using Tracer version 1.7.7 [28]. Tree Annotator version 1.10.4 was used to summarize the information from a sample of trees produced by BEAST on a single “target” tree to obtain the Bayesian maximum cluster credibility (MCC) tree. Next, we inferred the demographic history of different host H9N2 AIVs using the GMRF Bayesian Skyride plots.

### Virus replication in vitro

To detect replication of H9N2 AIVs in vitro, growth curves of viruses were obtained using MDCK, A549, HBE and CEF cells. Cells with 90% confluence were washed with phosphate-buffered saline (PBS) three times and incubated with viruses at one multiplicity of infection (MOI) for 2 h. Then, cells were cultured in DMEM containing 2% bovine serum albumin (BSA) (Sigma Aldrich, St. Louis, MO, USA), penicillin (100 units/mL), streptomycin (100 mg/mL), and TPCK-treated trypsin (1 μg/mL) at 37°C with 5% CO_2_. Supernatants were collected at 12, 24, 36, 48, 60, and 72 h post-infection (hpi). Viral titres were determined using MDCK cells and calculated with the Reed-Muench method. All experiments were performed in three independent biological replicates (*n* = 3).

### Receptor binding assay for viruses

To evaluate the receptor binding affinity of influenza virus strains CKSD2/22 (G57), CKSD3/21 (G57), CK201/20 (G118), and CK225/22 (G118), a solid-phase direct binding assay was performed using the following biotinylated sialic acid glycan receptors purchased from GlycoNZ, USA: 3'SLN and 6'SLN. An avian H5N1 influenza virus A/duck/Guangdong/212/2004 and a human H1N1 influenza virus A/Puerto Rico/8/1934 (H1N1) were used as typical avian and human tropism controls. Briefly, polystyrene universal binding microplates were coated with 100 μL of 10 μg/mL streptavidin (Solarbio, China) in carbonate buffer, and the plates were incubated at 4°C overnight. Next, the plates were washed three times with PBST (phosphate-buffered saline containing 0.05% Tween-20) and incubated with 3'SLN or 6'SLN, which were serially 2-fold diluted with PBS (0.78–100 ng/100 µL) at 4 °C for 24 h. Next, the plates were washed three times with PBST and incubated with monoclonal antibody against H9 subtype AIV at 4 °C for 5 h. After being washed three times with PBST, the plates were incubated with horseradish peroxidase (HRP)-conjugated goat anti-mouse antibody (Beyotime, China) at 4 °C for 2 h. Next, the plates were washed three times with PBST and incubated with TMB (3,3’,5,5’-Tetramethylbenzidine) (Beyotime, China) at room temperature for 10 min. Then, 0.05 mL of H_2_SO_4_ (0.5 mol/L) was added to the plates. The optical density at 450 nm was measured on a microplate reader.

### Pathogenicity of viruses in chickens

The pathogenicity of viruses in chickens was detected using four groups of eight 6-week-old specific-pathogen free (SPF) White Leghorn chickens, which were housed in isolators (150 cm × 85 cm × 100 cm). The chickens were administered 10^6^ EID_50_ of the corresponding viruses in a volume of 200 µL. At 3 days post infection (DPI), three birds in each group were randomly selected to be euthanized. The tracheas, lungs, spleens, intestines, kidneys, and brains were collected and viral titres were titrated in 9–11-day-old embryonated eggs. The viral titres were calculated by the Reed-Muench method. Total RNA was reverse transcribed into cDNA for absolute quantification of viral RNA. qPCR was performed with SupRealQ Purple SYBR Master Mix using G118-specific NP primers (forward 5′-GATGCCACATATCAGAGAACGAGAG-3′; reverse 5′-GCTCCATCACCATTGTTCCTATCC-3′) and G57-specific M primers (forward 5′-GGGACAGTGACCGCAGAAGG-3′; reverse 5′-TGCCTGTGGGACCGATGTTG-3′). Standard curves were generated for each genotype; the results were expressed as copy number/μL total RNA.

Oropharyngeal and cloacal swabs were collected at 1, 3, 5, 7, and 9 DPI and titrated in 9–11-day-old embryonated eggs. The sera were collected at 14 DPI, and the antibody titres were detected by the HI test. For histopathological examination, the lungs collected at 3 DPI were immersed in 4% paraformaldehyde, dehydrated, embedded in paraffin, and subjected to haematoxylin-eosin (HE) staining.

### Pathogenicity of viruses in mice

To assess the viral pathogenicity in mice, 32 6-week-old female BALB/c mice were randomly divided into four groups and housed in individually ventilated cages. Mice were anesthetized and intranasally inoculated with the corresponding viruses at a dose of 10^6^ EID_50_ in a volume of 50 μL. At 3 days post infection (DPI), three mice in each group were euthanized, and the brains, spleens, kidneys, and lungs were collected. Viral titres were titrated in 9–11-day-old embryonated eggs. Viral titres were quantified by the Reed-Muench method and expressed as EID_50_/mL. The remaining five mice in each group continued to be monitored, their body weights were recorded until 14 DPI, when the mice were then euthanized, and sera were collected. For histopathological examination, the lungs collected at 3 DPI were immersed in 4% paraformaldehyde, dehydrated, embedded in paraffin, and subjected to HE staining.

### Statistical analysis

One-way analysis of variance (ANOVA) with Turkey’s multiple comparison test was used to evaluate the statistically significant differences in H9N2 virus replication in vivo. All results were analyzed using Prism 9.5 (GraphPad Software Inc., San Diego, CA, USA) with a significance level of *p* < 0.05.

## Results

### Isolation and distribution of H9N2 viruses

**Figure 1.**
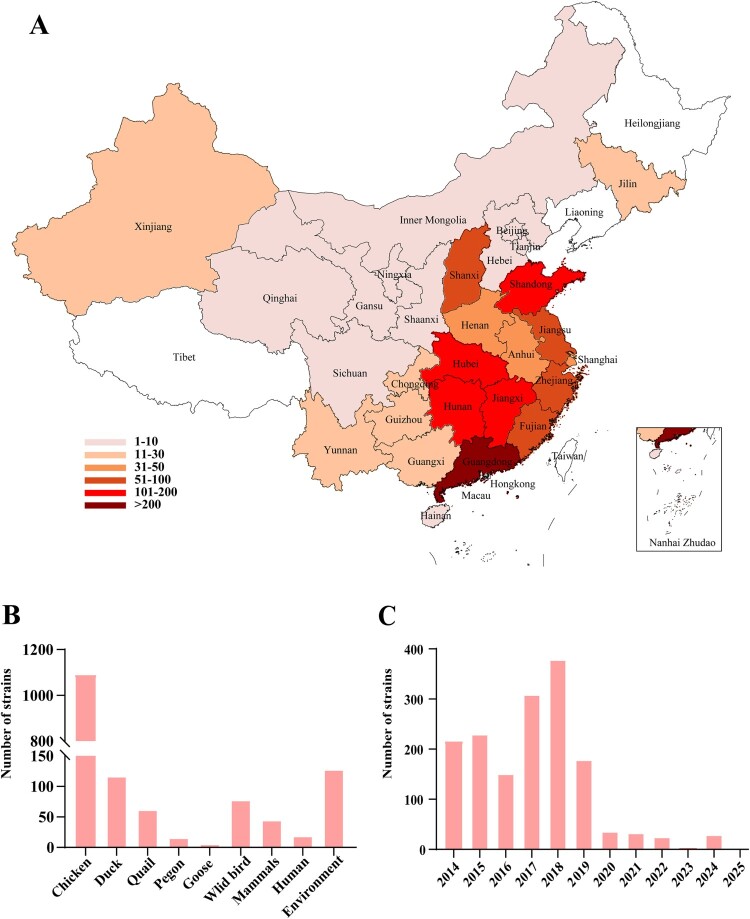
The geographical distribution, host, and temporal distribution analysis of 1,583 H9N2 AIVs in China in 2014–2025. (A)Geographic distribution. (B) Host distribution. (C) Temporal distribution.

To better understand the evolution and prevalence of H9N2 viruses in China, we collected high-quality whole-genome sequences of Chinese H9N2 viruses (*n* = 1,583) from the GISAID and GenBank Flu databases from 2014 to 2025 (Table S1). The sequences cover most regions of China, including 29 provinces, municipalities, and autonomous regions (Figure 1(A)). In the host distribution analysis, 1,114, 117, 6, 62, 17, 80, 128, 47, and 12 strains were selected from chickens, ducks, geese, quail, pigeons, wild birds, environment, humans, and other mammals (including dogs, cats, minks, pigs, and foxes), respectively (Figure 1(B)). In the time distribution analysis, 216, 230, 151, 309, 379, 179, 36, 33, 25, 3, 29, and 1 strain were selected from 2014, 2015, 2016, 2017, 2018, 2019, 2020, 2021, 2022, 2023, 2024, and 2025, respectively (Figure 1(C)). In addition to publicly available genomic sequences, eight H9N2 avian influenza viruses were isolated from poultry samples in Guangdong and Shandong provinces during 2020–2022 in this study. Detailed information regarding these eight isolates, including collection location, host origin, sampling year, and accession numbers, is summarized in Table S2. Genotyping analysis indicated that these newly obtained strains belonged to the two predominant genotypes currently circulating in China, specifically two strains of genotype G57 and six strains of genotype G118. Representative strains were further selected from these eight isolates for subsequent phylogenetic analysis, in vitro replication assays, and in vivo pathogenicity assessment, to characterize the biological phenotypes of recently circulating dominant H9N2 strains in China.

### Phylogenetic analysis of HA and NA genes

**Figure 2.**
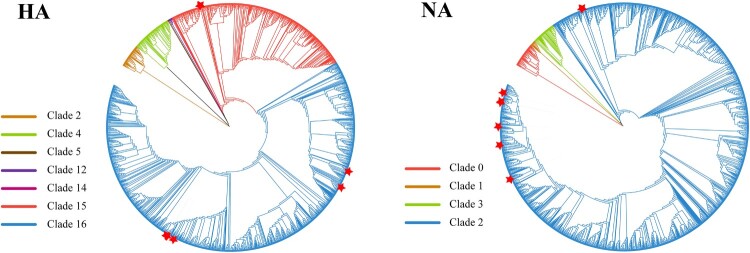
Phylogenetic analysis of HA and NA genes of H9N2 AIVs. (A) Maximum-likelihood (ML) phylogenetic tree of the HA gene of H9N2 AIVs. (B) Maximum-likelihood (ML) phylogenetic tree of the HA gene of H9N2 AIVs. The red five-pointed star represents the isolated strain in our laboratory.

Multiple sequence alignment and phylogenetic analysis were used to study HA and NA gene sequences of H9N2 viruses isolated in China from 2014 to 2025. We constructed a phylogenetic tree for each gene by using the first 20th-percentile cut-off values, manually merged the branches through their branch posteriors, and reported classification if necessary. According to the ML tree of the HA gene, H9N2 viruses have evolved into seven different genetic clades (Clades 2, 4, 5, 12, 14, 15, and 16). The 394 strains clustered in Clade 5 (*n* = 1), Clade 12 (*n* = 4), Clade 14 (*n* = 1), and Clade 15 (*n* = 388), which originated from A/chicken/Beijing/1/94 (BJ/94-original). 48 strains were clustered in Clade 2 to form the Korean-like lineage, and 83 strains were clustered in Clade 4 and formed a G1-like branch. 1066 strains were clustered in Clade 16, which emerged in April 2012. Among the 8 H9N2 AIVs isolated in this study (Table S2), 2 strains were clustered in Clade 15, and 6 strains were clustered in Clade 16 (Figure 2). Clades 15 and 16 replaced other clades as the dominant clades.

The NA clades were mainly clustered in 4 clades (Clades 0–3) according to the previous classifications of H9N2 viruses in China. At present, NA genes are still distributed in 4 different sub-branches. 1,463 strains are clustered in Clade 1 (*n* = 9) and Clade 2 (*n* = 1,482), which evolved from the BJ-94-like lineage, and Clades 0 and 3 feature 51 strains each. However, the isolates in this study were clustered in Clade 2, which became the dominant branch in China after 2010 (Figure 2).

### Phylogenetic analysis of internal genes

To solve the problem of confusion in the internal gene naming of the H9N2 AIVs, previous studies have named the internal genes using numbers based on phylogenetic development. All six internal segments are divided into 42 separate clades. We analysed the internal genes of H9N2 AIVs that have been found in China in the past 10 years, and only 21 clades of them were found. According to the phylogenetic tree of the PB2 gene, 1561 strains were distributed in 4 branches, of which 10 strains were clustered in Clade 0, and 7 strains were clustered in Clade 6. These clades were formed by the BJ-94 and F/98 lineages. About 85 strains belonged to Clade 7, and the viruses in this branch were mainly isolated from quails and other wild birds. The remaining 1,488 strains belonged to Clade 8, including the most prevalent strain since 2010, and the isolates found in this study were also distributed in this branch.

The phylogenetic tree of the PB1 gene also formed four clades. Among the 1,591 strains, 1,511 PB1 genes were clustered in Clade 5, which evolved from the SF/98-like lineage and has been the most common branch in China since 2010. The PB1 gene of the strains isolated in our laboratory was also clustered in Clade 5. The remaining strains were clustered in Clade 0 (*n* = 13), Clade 2 (*n* = 43), and Clade 3 (*n* = 24).

All strains of the PA gene were assigned to 4 clades: Clade 3 (*n* = 9), Clade 5 (*n* = 60), Clade 6 (*n* = 6), and Clade 7 (*n* = 1,516), while the 8 laboratory isolates were clustered in Clade 7. There were 21 and 34 NP genes clustered in Clades 1 and 4, respectively. 1,506 strains belonged to Clade 6 and evolved from the SF/98 lineage. The NP genes of the laboratory isolates in this study were also clustered in this clade.

The M genes were clustered into 4 clades: Clade 0 (*n* = 15), Clade 1 (*n* = 59), Clade 2 (*n* = 2), and the currently prevalent Clade 3 (*n* = 1,515), which originated from the G1-like lineage. The 8 isolates in this study were all clustered in Clade 3. For the NS gene, the phylogenetic tree showed that all strains were clustered in only Clade 0 (*n* = 85) and Clade 7 (*n* = 1,506), which originated from the Y439-like lineage and BJ-94-like lineage, respectively. The NS genes of the 8 H9N2 AIVs isolated in this study were all clustered in Clade 7 (Figure S1).

### Genotypic analyses

**Figure 3.**
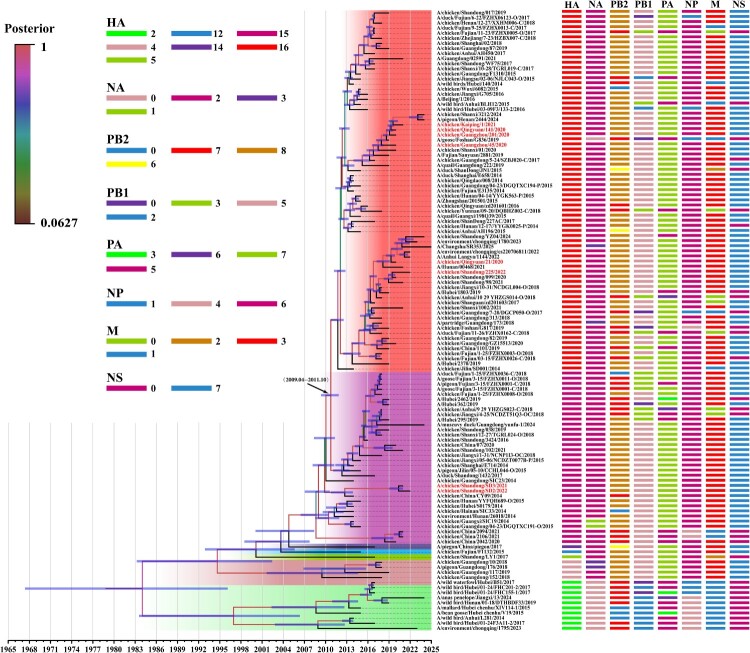
Molecular clock phylogenetic analysis of H9N2 AIVs in 2014–2025. Phylogeny of the HA gene and genotype evolution of H9N2 AIVs. The phylogenetic tree of HA gene using BEAST (version 1.10.4) under the exponential coalescent tree prior model, “GTR + F + G4” substitution mode, and a “uncorrelated relaxed clock” model. Purple node bars represent 95% credible intervals of lineage divergence times. The eight segments represented by horizontal bars are, from top to bottom of the virion, PB2, PB1, PA, HA, NP, NA, M, and NS.

**Figure 4.**
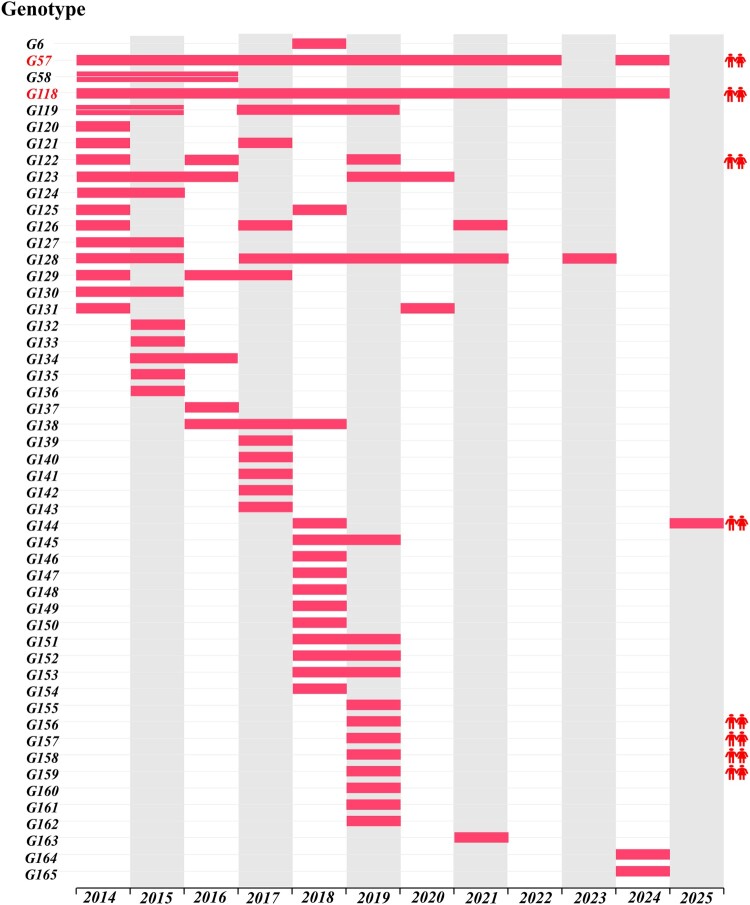
Timeline of emergence of different genotypes of H9N2 viruses in 2014–2025. Red bars represent duration of circulation of H9N2 viruses during 2014–2025. The red cartoon symbol indicates that this genotype is associated with human infections.

We found 52 genotypes in all eight segments of 1,591 H9N2 viruses isolated from 2014 to 2025, including 48 newly discovered genotypes G118–G165 and 4 previously reported genotypes (G6, G57, G58, and G68) (Figure 3 and Table S1). Among the 48 newly defined genotypes, G118, G119, G123, G128, and G138 were continuously observed for more than 3 years and were considered the main genotypes, while the others were considered temporary genotypes. From the perspective of time distribution, the main genotypes appeared between 2014 and 2016, and all genotypes observed in 2017–2020 were transient genotypes. After 2020, three transient genotypes were isolated: G163, G164, and G165. The genotypes in infected patients included G57, G118, G122, G156, G157, G158, and G159 (Figure 4).

Among the 1,591 strains examined in this study, 356 strains belonged to the G57 genotype, which accounted for 22.37%. 1,013 strains belonged to the newly discovered G118 genotype, which accounted for 63.67%, and the remaining 48 genotypes only accounted for 13.96%. Of the 8 isolates obtained in this study, 2 belonged to the G57 genotype, and the remaining 6 belonged to the newly defined G118 genotype. Compared with G57, the newly defined G118 genotype differed in only the HA gene. The HA of G57 was from Clade 15, while the HA of G118 was from Clade 16. The remaining genes were from the same clades (the PB2 gene from Clade 8, PB1 from Clade 5, PB2 from Clade 8, PB1 from Clade 5, PA gene from Clade 7, NP gene from Clade 6, M gene from Clade 3, NS gene from Clade 7, and NA gene from Clade 2). Currently, the G57 and G118 genotypes are the two dominant genotypes of H9N2 AIVs in China.

### Molecular characteristics of HA protein

**Figure 5.**
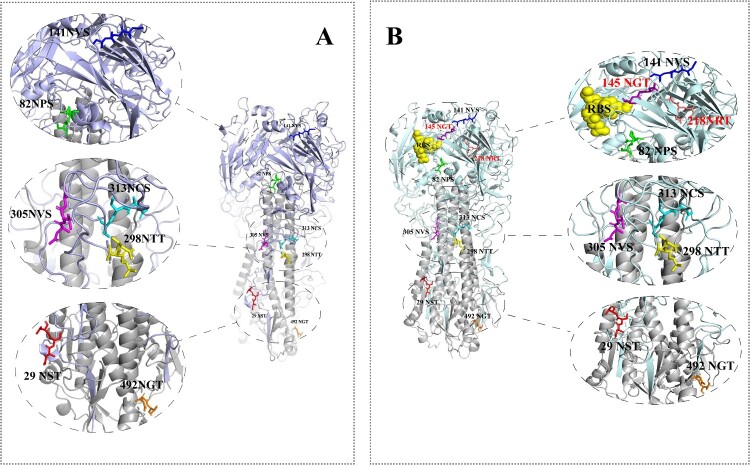
The spatial locations of substituted amino acids on the HA 3D structure. The structure of the HA protein trimer was drawn with PyMOL. (A) shows the detailed structure of the HA globular head of A/chicken/Jiangsu/WJ57/2012 (Vaccine strain); The HA1 and HA2 sections were shown in light purple and grey colours, respectively. (B) shows the detailed structure of the HA globular head of A/chicken/Anhui/LH99/2017 (Representative circulating strain); The HA1 and HA2 sections were shown in light green and grey colours, respectively. The receptor binding site (RBS) was yellow. The 29-, 82-, 141-, 145-, 218-, 298-, 305-, 313- and 492-glycosylation sites on HA are marked in Red, green, blue, purple, orange, yellowish, light purple, cyan and brown, respectively (H9 numbering). The 145- and 218-glycosylation sites of representative prevalent strains differ from that of vaccine strains, and the names of the 145- and 218-glycosylation sites in Figure 7B are marked in red.

The HA protein variation of H9N2 AIVs in the past 10 years was examined by predicting their potential glycosylation sites in the 1,591 H9N2 AIVs isolated in China from 2014 to 2025 and the 8 H9N2 AIVs isolated in this study. The results showed that all 8 isolates had 7 potential glycosylation sites: 2 in the head of the HA protein structure and 5 in the stem (29-NST, 141-NVS, 298-NTT, 305-NVS, 313-NCS, and 492-NGT) (H9 numbering). 313-NCS was not present in the vaccine strains A/chicken/Guangdong/SS/94 (SS/94) and A/chicken/Shanghai/F/98 (F98), and the 218-NRT site was absent. The results were consistent with the prediction for the vaccine strain A/chicken/Jiangsu/WJ57/2012 (WJ57) (Figure 5 and Table S3).

Among the HA proteins of the 1,591 H9N2 AIVs, 109 strains had 218-NRT potential glycosylation sites that were not found in the vaccine strain WJ57, and 6 strains had potential 145-NGT glycosylation sites near RBS. However, this site was not detected in the vaccine strains SS/94, F98, and WJ57. The results showed that sialic acid receptor binding sites 183 and 226 of the HA protein of the 8 isolates in this study were an N and L residue, respectively. The 190 T mutation of HA protein was obtained in CK201/20, CK45/20, CK141/20 and CK1/21. Except for CKSD2/22 and CKSD3/21, the other 6 isolates contained 192R mutation (Table 1). The amino acid sequence of the HA protein cleavage site of all isolates was PSRSSR↓GLFGAI, which is a molecular characteristic of LPAIV. Compared with the cleavage site sequence of vaccine strains (SS/94, F98, and SD/6/96), the second amino acid mutated from alanine (A, non-polar amino acid) to serine (S, polar amino acid).

**Table 1.** Key amino acid sites of H9N2 viruses used in this study.

Viruses	HA							PB2				PB1	PA	NP
	183	190	192	226	195	253	292	588	591	627	701	577	356	434
A/chicken/Guangdong/SS/94*	N	A	T	Q	/	/	/	/	/	/	/	/	/	/
A/chicken/Shanghai/F/98*	N	A	T	Q	D	D	V	A	Q	E	D	K	K	E
A/chicken/Shandong/6/96*	N	A	T	Q	D	D	I	A	L	E	D	K	K	E
A/chicken/Jiangsu/WJ57/2012*	N	V	T	L	D	D	V	A	Q	E	D	K	R	E
A/chicken/Qingyuan/21/2020	N	V	R	L	D	D	V	V	Q	E	D	K	R	E
A/chicken/Guangzhou/201/2020	N	T	R	L	D	D	V	V	Q	E	D	K	R	E
A/chicken/Guangzhou/45/2020	N	T	R	L	N	N	V	V	Q	E	D	K	K	E
A/chicken/Qingyuan/141/2020	N	T	R	L	D	D	V	V	Q	E	D	K	R	E
A/chicken/Kaiping/1/2021	N	T	R	L	D	D	I	V	K	E	D	K	R	E
A/chicken/Shandong/SD3/2021	N	V	T	L	D	D	V	A	Q	E	D	K	K	E
A/chicken/Shandong/SD2/2022	N	V	T	L	D	D	V	A	Q	E	D	K	K	E
A/chicken/Shandong/225/2022	N	V	R	L	D	D	V	V	Q	E	D	K	R	E

Note: “*” denotes the vaccine strain of H9N2. The amino acid positions of HA protein were numbered according to the H3 subtype numbering system, and the amino acid position of PB2, PB1, PA and NP proteins were numbered according to H9N2 subtype numbering system. “/”: The sequence was not found in NCBI and GISAID.

### Spatial dynamics of geographic dispersal

**Figure 6.**
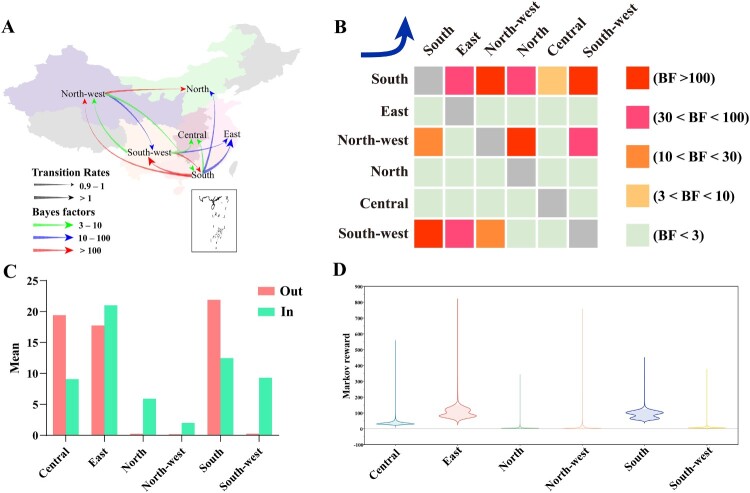
Phylogeographic analysis of H9N2 AIVs was determined by Bayesian phylogeographic inference of HA in China in 2017–2022. (A) Spatiotemporal spread of H9N2 AIVs; curves show the inter-region virus transitions statistically supported with Bayes factor (BF) >  3; curve widths represent transition rate values; curve colours represent corresponding statistical BF for each transition rate. (B) The heatmaps display the level of BF for each of the transition pathways considered for H9N2 AIV analyses. (C) Histograms of the total number of region transitions. (D) Density distribution of the total time spent in a particular region population (Markov rewards).

To understand the spatial dynamics of H9N2’s geographical spread in China, we estimated its prevalence using BSSVS analysis. A total of 197 H9N2 strains were included in the BSSVS spatial dynamics analysis, including 74 strains in East China, 30 strains in Central China, 8 strains in North China, 3 strains in Northwest China, 69 strains in South China, and 13 strains in Southwest China. Considering the complex ecology of AIVs in China, the country was divided into 6 geographical regions (East, Central, Northeast, Northwest, South, and Southwest China). Our Bayesian phylogeographic analysis indicated 12 migration links in the spread of H9N2 AIVs in China, including 5 transmission routes from East China. There were 4 paths with decisive support (BF > 100): South to Southwest China (BF = 38,425.4), Northwest to North China (BF = 19,210.5), Northwest to South China (BF = 251.9), and South to Northwest China (BF = 188.8) (Figure 6(A, B)).

In addition, several routes to Central China and East China were identified. We observed that South China was the main epicentre and mediated four obvious migration routes of H9N2 AIVs with migration rates greater than 0.9, including routes from South China to East China (migration rate = 2.021), from South China to Southwest China (migration rate = 1.125), from South China to Northeast China (migration rate = 0.952), and from South China to Northwest China (migration rate = 0.937). Markov jumps reflect the phenomenon of the virus migrating outward from South China, East China, and Central China and inward to East China, South China, and Southwest China (Figure 6(C, D)). These results indicated that the South, Southwest, and Northwest regions played key roles in the spread of H9N2 AIVs in China from 2017 to 2022.

### Host transition

Due to the wide host range of H9N2 AIVs, they can infect a variety of poultry species. Studies have shown that H9N2 AIVs that initially infected chickens and ducks in mainland China have been transmitted by quails [3]. To evaluate the interspecies transmission of H9N2 AIVs in China in the past five years, we used chickens, ducks, geese, pigeons, and wild birds as hosts to analyse the interspecific evolution of H9N2 AIVs using the BSSVS program. 117 strains were selected for host transition analysis, including 71 strains from chickens, 36 strains from ducks, 2 strains from geese, 4 strains from pigeons, and 4 strains from wild birds.

**Figure 7.**
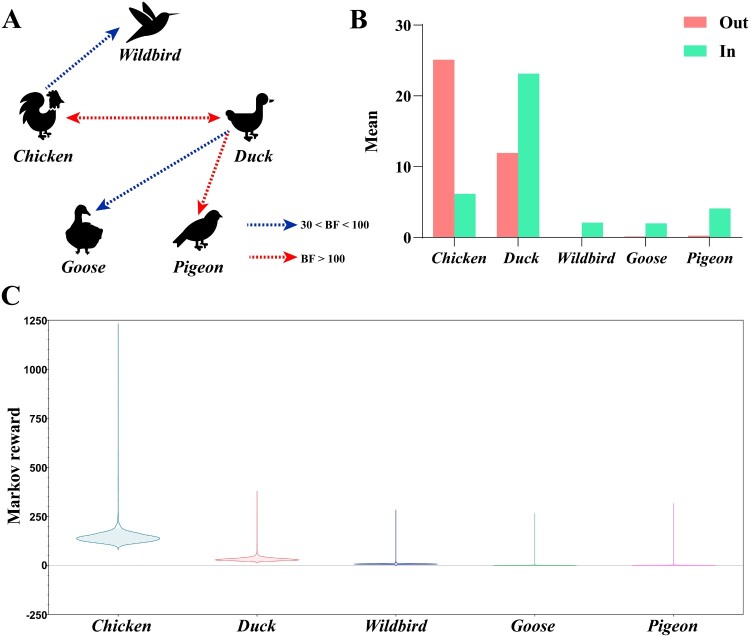
Host transition of H9N2 AIVs. (A, B) Host transition of H9N2 AIVs in China. Only statistically supported migrations with BF > 3 are shown. (C). Density distribution of the total time spent in a particular host population (Markov rewards).

The results showed that there were 5 viral transmissions with statistical significance (BF > 3). One of them involved a virus transformation from poultry (chickens) to wild birds (BF = 40.6). There were 4 virus conversions between poultry: from chickens to ducks (BF = 58,707.5), from ducks to pigeons (BF = 4,889.3), from ducks to chickens (BF = 317.5), and from ducks to geese (BF = 27.09). In addition, a high average ratio was observed, which further indicates the transformation of these viruses (Figure 7(A)). The total number of host-state transitions obtained from Markov jump counts analysis showed that chickens had the highest Markov reward time, followed by ducks, wild birds, geese, and pigeons (Figure 7(B, C)). A higher Markov reward time indicates longer sustained viral persistence and greater cumulative prevalence within a host group, consistent with the largest number of H9N2 isolates recovered from chickens and ducks. These results indicate that H9N2 AIVs have maintained a high prevalence in chickens and ducks in China and are highly likely to spread from these birds.

### Population dynamics in different hosts

**Figure 8.**
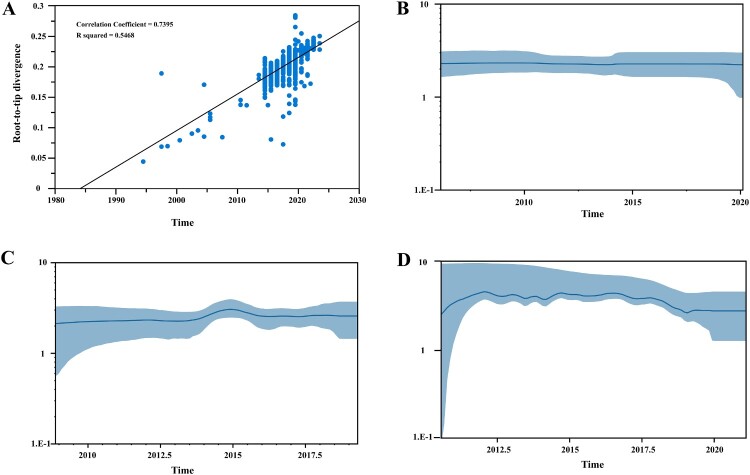
Evolutionary dynamics of H9N2 AIVs. (A) Analysis of root-to-tip divergence against sampling date for HA gene segment. A GMRF Bayesian Skyride analysis of HA gene of H9N2 AIVs from wild birds. (B), waterfowl from poultry (C), and terrestrial birds from poultry. (D) to display changes in the effective population size over time. The solid blue line indicates the mean value, and the shaded blue area represents the 95% highest posterior density of genetic diversity estimates.

We also explored the history of population dynamics of H9N2 AIVs from different hosts in China. Because of its wide host range and few available sequences from some hosts, we focused on its main hosts, poultry, for further study. Three groups were examined: wild birds, waterfowl poultry, and chickens. Using root-to-tip regression, our temporal structure analysis revealed that H9N2 AIVs showed a clock-like structure (correlation coefficient = 0.739; R^2^ = 0.546) (Figure 8(A)).

To illustrate the effective population size of these viruses, a Bayesian skyline coalescent model was constructed. The number of H9N2 AIVs in wild birds was relatively constant during the observation period (Figure 8(B)). From 2014 to 2016, the population size of H9N2 AIVs in waterfowl poultry showed a significant expansion and then gradually stabilized (Figure 8(C)). The population size of H9N2 AIVs in Galliformes poultry gradually expanded in 2011 and then continued to fluctuate from 2012 to 2018 (Figure 8(D)).

### Replication kinetics in different cells

**Figure 9.**
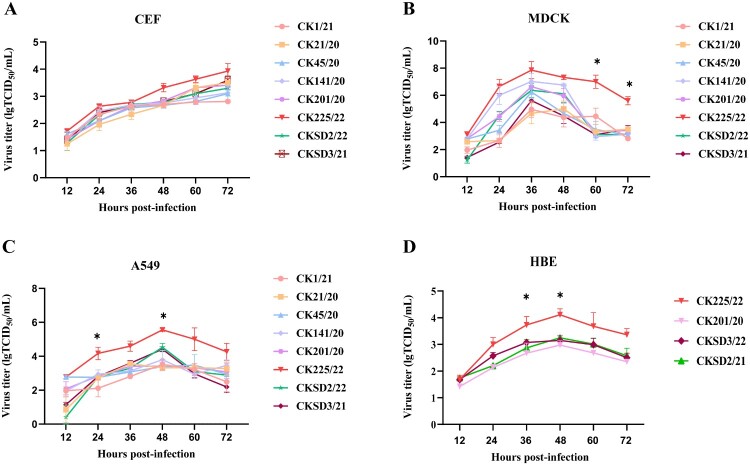
Growth kinetics of H9N2 viruses in MDCK, A549, CEF, HBE cells. (A) CEF cells. (B) MDCK cells. (C) A549 cells. (D) HBE cells. Viral titres in supernatants were measured at indicated time points by TCID_50_/mL. The data of each time point indicates the mean ± standard deviation of three independent experiments. ***p < 0.001, **p < 0.01, *p < 0.05.

To study the replication kinetics of two prevalent genotypes of H9N2 AIV (G57 and G118) in China, growth curves were obtained using MDCK, A549, and CEF cells for the 8 viruses isolated in this study (G57:CKSD2/22, CKSD3/21; G118:CK1/21, CK21/20, CK45/20, CK141/20, CK201/20, and CK225/22). The replication ability of four representative viruses CKSD2/22 (G57), CKSD3/21 (G57), CK201/20 (G118), and CK225/22 (G118) in human bronchial epithelial (HBE) cells was also evaluated. The virus replicated efficiently in CEF cells, with viral titres of 0.97–4.25 lgTCID_50_/mL, and the highest titre of 4.25 lgTCID_50_/mL was reached at 72 hpi. Notably, the titre of CK225/22 (G118) in CEF cells at 48 hpi was higher than that of the other viruses (*p* < 0.01) (Figure 9(A)).

In MDCK cells, the viral titres of the 8 viruses ranged from 1.83 to 5.67lgTCID_50_/mL, and the peak titre appeared at 36 hpi. Importantly, the titres of CK225/22 (G118) in MDCK cells at 60 and 72 hpi were higher than those of other viruses (*p* < 0.001) (Figure 9(B)). In A549 cells, the virus titres of eight viruses ranged from 0 to 5.77 lgTCID_50_/mL. Aside from CK21/20 (G118), which reached its peak titre at 36 hpi, the other 7 viruses reached peak titres at 48 hpi. Notably, the peak titre of CK225/22 (G118) in A549 cells was higher than that of the other 7 viruses (*p* < 0.01) (Figure 9(C)). In HBE cells, the four strains showed a similar upward trend in viral titres, reaching a peak between 36 and 48 hpi. Notably, the viral titre of CK225/22 was significantly higher than those of CK201/20, CKSD3/22, and CKSD2/21 at 36 and 48 hpi (*p* < 0.05), indicating that it has good replication ability in human bronchial epithelial cells (Figure 9(D)).

### Receptor binding assay for viruses

**Figure 10.**
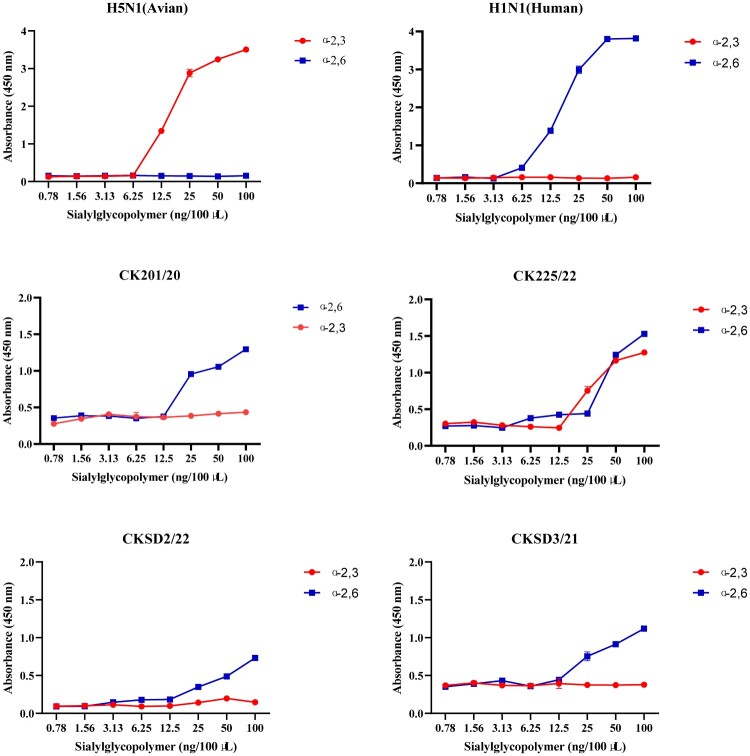
Receptor binding properties of the 4 H9N2 influenza viruses. The solid-phase binding assays of H5N1(DK212), H1N1(PR8/34), CKSD2/22, CKSD3/21, CK201/20, and CK225/22 with two different sialylglycopolymers (3'SLN coloured in red and 6'SLN coloured in blue).

To characterize the receptor-binding affinity of the four H9N2 AIVs, a solid-phase binding assay was performed. The results showed that H5N1 DK212 virus only bound to α-2,3-linked sialylglycopolymers (avian receptors), and H1N1 PR8/34 virus only bound to α-2,6-linked sialylglycopolymers (human receptors). The CK201/20 (G118), CK225/22 (G118), CKSD2/22 (G57) and CKSD3/21 (G57) exhibited dual receptor-binding tropism, and preferentially bound to α-2,6-linked sialylglycopolymers (human receptors) (Figure 10).

### Replication and pathogenicity in chickens

**Figure 11.**
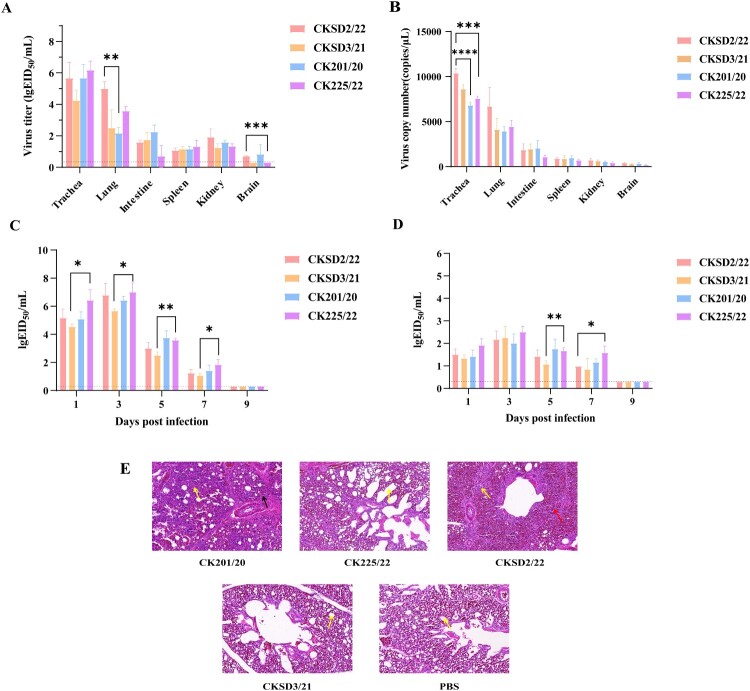
Replication and pathogenicity of the H9N2 viruses in chicken. (A) Viral titres in various organs of infected chickens. The dashed line indicates the lower limit of detection. Data are presented as mean ± SD. Statistical analyses were performed using one-way analysis of variance (ANOVA) using the Turkey’s multiple comparison test. ***p < 0.001, **p < 0.01, *p < 0.05. (B) Replication of the four H9N2 viruses from G57 and G118 in chickens. The organs were collected at 3 dpi for qRT-PCR detection. The data displayed represent the average titre ± standard deviation (SD) of 3 chickens in each group. Statistical analyses were performed using multiple t-tests. ***p < 0.001, **p < 0.01, *p < 0.05. (C) Throat viral shedding after infection with four H9N2 AIVs. (D) Cloacal viral shedding after infection with four H9N2 AIVs. (E) Lung histopathological section of SPF chicken (200×, H&E staining). Yellow arrows indicate tertiary bronchi; red arrows indicate extensive vascular congestion in the interstitium; green arrows indicate necrosis of a small number of bronchial epithelial cells with karyopyknosis and karyolysis; black arrows indicate marked inflammatory cell infiltration.

We selected two strains from each of the G57 and G118 genotypes to study the replication and virulence of H9N2 AIVs in chickens: CKSD2/22 (G57), CKSD3/21 (G57), CK201/20 (G118), and CK225/22 (G118). At 3 DPI, gross pathological examination revealed moderate to severe lesions in the trachea and lungs, while only mild lesions were observed in the spleen, brain, intestine, and kidney (Figure S2). The four viruses could replicate in the trachea, lungs, cecum, and kidneys of chickens, but the replication efficiency of CKSD2/22 in the lungs was significantly higher than that of CK201/20. The viral titres of the four viruses in the tracheas were higher than those in the lungs, indicating that the viruses can replicate in the upper respiratory tract. Low viral titres were detected in the brain of chickens infected with CKSD2/22 and CK201/20, while no virus was detected in the brain of chickens infected with CKSD3/21 and CK225/22 (Figure 11(A)). To evaluate the tissue range and replication ability of the selected four H9N2 viruses in chickens, the viral loads in trachea, lung, intestine, kidney, spleen, and brain were quantified at 3 DPI. Overall, the four viruses showed a consistent distribution pattern, and the viral load in the respiratory tract (trachea and lung) was always higher than that in the intestine and parenchymal organs (kidney and spleen). Notably, the viral load in the intestine was significantly lower than that in the trachea and lungs, but still higher than that in the kidneys and spleen. Specifically, the tracheal viral load of CKSD2/22 was significantly higher than that of CK201/20 (*p* < 0.0001) and CK225/22 (*p* < 0.001), and there was no statistical difference between the other strains. In contrast, the viral loads in the kidneys and spleen were generally low, and there was no significant difference between the four viruses (*p* > 0.05). In the brain, only trace amounts of viral nucleic acid were detected (Figure 11(B)). These results indicated that both genotypes of H9N2 AIVs replicated well in the respiratory organs of chickens and had tissue tropism.

Next, we determined viral shedding of four H9N2 AIVs in chickens. CKSD3/21, CKSD2/22, CK/201/22, and CK225/22 viruses were detected in oropharynx swabs at 1, 3, 5, and 7 DPI, and the titres were 0.98–5.75 l gEID_50_/mL, 0.98–7.75 lgEID_50_/mL, 0.98–6.75 lgEID_50_/mL, and 1.50–7.75 lgEID_50_/mL. The peaks were reached at 3 DPI. The peak virus titre of CK225/22 was the highest at 7.75 lgEID_50_/mL, while the peak virus titre of oropharynx swabs of CKSD3/21 was the lowest at 5.75 lgEID_50_/mL (Figure 11(C)).

In inoculated chickens, the virus was detected in the cloaca of the CKSD3/21, CKSD2/22, CK/201/22, and CK225/22 groups at 1, 3, 5, and 7 DPI with titres of 0.98–2.25 lgEID_50_/mL, 0.98–2.50 lgEID_50_/mL, 0.98–2.50 lgEID_50_/mL, and 1.25–2.75 lgEID_50_/mL, respectively. The peaks were reached at 3 DPI. The peak virus titre of CK225/22 was the highest at 2.75 lgEID_50_/mL, while the peak virus titre of the cloaca swab of CKSD3/21 was the lowest at 2.25 lgEID_50_/mL (Figure 11(D)). The sera were collected at 14 DPI and titrated by HI assay (Table S4). The results showed that all sera had positive results, and the HI titres were 1:2560–1:10240. H&E staining showed that there were pathological changes in the lungs of chickens infected with CKSD2/22 and CK201/21, such as inflammatory cell infiltration, haemorrhage, and the pulmonary interstitium was significantly thickened, accompanied by extensive inflammatory cell infiltration (mainly lymphocytes and heterophils), and the normal airspace structure was lost, resulting in obvious pulmonary parenchymal consolidation. The lung parenchyma structure of CK225/22 and CKSD3/21 infected groups was basically normal. No shedding and necrosis of mucosal epithelial cells were observed. The tertiary bronchus maintained clear structural integrity without and no obvious atrophy or collapse (Figure 11(E)).

### Pathogenicity in mice

**Figure 12.**
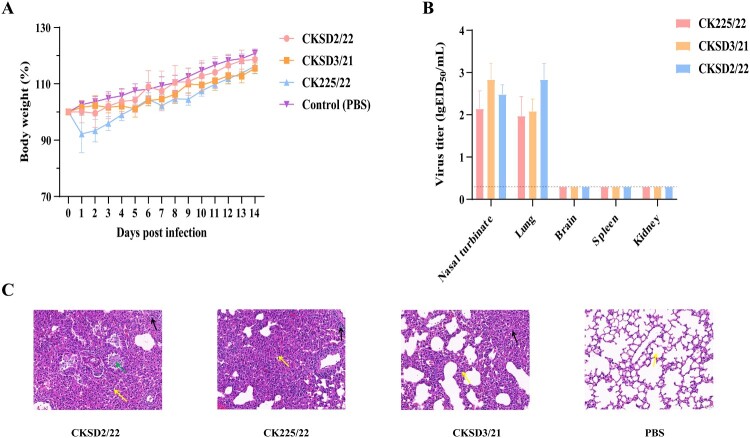
Replication and pathogenicity of the H9N2 viruses in mice. (A)Body weight changes in mice post-infection. (B) Viral titres in various organs of infected mice. The dashed line indicates the lower limit of detection. Data are presented as mean ± SD. Data are presented as mean ± SD. Statistical analyses were performed using multiple t-tests. ***p < 0.001, **p < 0.01, *p < 0.05. (C) Mice lung histopathologica section, H&E stain, 200× magnification. Yellow arrows indicate tertiary bronchi; red arrows indicate extensive vascular congestion in the interstitium; green arrows indicate necrosis of a small number of bronchial epithelial cells with karyopyknosis and karyolysis; black arrows indicate marked inflammatory cell infiltration.

To assess the potential threat of the H9N2 viruses to mammals, we assessed the replication and virulence of three H9N2 viruses from different genotypes in mice: CK225/22 (G118), CKSD3/21 (G57), and CKSD2/22 (G57). At 2 DPI, the body weight of CK225/22 infected mice decreased significantly, while the other two H9N2 AIV infected groups had no obvious clinical symptoms. Notably, none of the tested viruses led to lethal infection in mice (Figure 12(A)).

At 3 DPI, the mice infected with CK225/22 showed ruffled fur and lethargy. The virus was detected in the nasal turbinate, lung, brain, spleen, and kidney. Viral titres were only detected in the nasal turbinate and lungs of infected mice, and no virus was detected in other organs (brain, kidney, and spleen). The titres of all three strains in the nasal turbinate were higher than those in the lungs. The viral titres of CK225/22, CKSD3/21, and CKSD2/22 in the nasal turbinate were 2.14–2.83lgEID_50_/g/mL. The virus titres of CK225/22, CKSD3/21, and CKSD2/22 in the lungs were 1.97­2.83 lgEID_50_/g/mL (Figure 12(B)). All sera showed positive results, and the titres were 1: 320–1:1280 (Table S5). These results indicated that viral replication is restricted to the respiratory tract (nasal turbinate and lungs) without detectable dissemination to systemic organs (brain, kidneys, spleen). H&E staining showed that significant lung injury occurred in all infected mice. In the CKSD2/22 infection group, the normal lung tissue structure was severely damaged, and the parenchymal structure was almost completely disappeared. The alveolar wall was significantly thickened, the alveolar structure was lost, and the interstitial and alveolar spaces were extensively infiltrated by inflammatory cells, accompanied by interstitial congestion, edema, and perivascular inflammation. In the CKSD3/21 group, obvious alveolar wall thickening, focal alveolar atrophy and collapse were observed, and moderate levels of inflammatory cell infiltration were observed in the affected area. The whole lung structure of CK225/22 group was also abnormal, with multifocal alveolar atrophy and collapse, obvious thickening of alveolar wall, and obvious inflammatory cell infiltration in lung parenchyma. These changes were more serious than those in the CKSD3/21 group (Figure 12(C)).

## Discussion

In this study, 48 new genotypes were identified by phylogenetic and genotypic evolution analysis of H9N2 viruses from different host sources in China from 2014 to 2025. The H9N2 AIV of G57 and G118 genotypes continues to be prevalent in chickens and ducks and is transmitted to other hosts through 12 distinct migration pathways, becoming the main epidemic genotypes in China. Among them, the South China region is the main transmission centre. H9N2 viruses of G57 and G118 genotypes preferentially bind to human-type receptor, replicate efficiently in MDCK, A549 and HBE cells, and cause pathology in mice. They pose an increasing potential threat to public health. Therefore, the epidemiological and aetiological surveillance of H9N2 AIV should be strengthened.

Due to frequent gene exchange between different hosts, multiple genotypes are formed. Before 2013, 117 genotypes were identified, including 45 major genotypes and 72 transient genotypes, indicating three important time points for the evolution of H9N2 AIVs in China: 2000, 2006, and 2010 [19]. The chicken-derived F/98 viruses have become the dominant lineage, and the other lineages have gradually decreased. Subsequently, in 2010, the G57 genotype became the only dominant genotype in the chicken flock, followed by the emergence of a new H7N9 reassortant containing six H9N2 internal genes [19]. Our findings showed that 48 new genotypes were formed in 2014–2025, and the new G118 genotype identified in this study, together with the G57 genotype, constitute the main epidemic genotypes in China in the past 10 years. The HA gene of G57 genotype was from Clade 15, while the HA gene of G118 genotype was from Clade 16. Studies have shown that the antisera against Clade 16 strains reduced cross-reactivity with the Clade 15 viruses in the HI assays, and the antigenicity has changed [20]. In addition, although many genotypes defined as “major genotypes” in previous studies were not detected in this study, new genotypes are emerging, indicating that H9N2 AIVs are constantly reassorting and mutating. However, most genotypes are temporary in the process of genetic evolution and cannot be maintained stably in the population.

The leucine residue at position 226 of the HA protein enhances the ability of the virus to bind to the α-2,6 sialic acid receptors and plays an important role in the transmission of H9N2 in ferrets [29]. Most of the H9N2 AIVs carrying HA Q226L, I155 T, and H183N mutations preferentially bind to human-like receptors [30]. For HA, it contains 183N, 190 T, 192R, and 226L mutations, which are associated with increased binding to 2,6 SLN or increased airborne transmission [31]. The H9N2 AIV that has been prevalent in recent years shows dual tropism, and its ability to bind to human-like receptors is higher than that of avian-like receptors [32, 33]. Our results showed that current H9N2 AIVs obtained 183N and 226L mutations and showed dual receptor binding tropism , preferentially binding to human-like receptors. These findings are consistent with previous studies. CK225/22 virus showed strong binding affinity to human- and avian-like receptors, while the other three viruses preferentially bind to human-like receptors. This discrepancy may be involved with the 190 V and 192R mutation at HA protein of CK225/22. The disappearance of the 218 glycosylation site combined with the appearance of the 313 glycosylation site changed the antigenicity of the virus [34]. Our results showed that current H9N2 AIVs have such molecular characteristics. However, this mutation did not appear in the vaccine strains. Therefore, attention should be paid to these changes when updating the H9N2 vaccine strain.

The H9N2 virus was first prevalent in the Pearl River Delta region of southern China and gradually spread to 15 provinces [19]. From 2010 to 2013, the eastern coastal areas became an important focus of H9N2 infection. The results of this study showed that South China was the transmission centre of H9N2 virus in China in the past five years, and South China, Southwest China, and Northwest China were related to the multiple introduction of H9N2 virus in other geographical regions of China. This indicates that the transmission route of H9N2 virus became complicated. These changes may be related to environmental and commercial factors such as warm climate, bird migration, developed poultry industry and trade [35, 36].

Although inactivated vaccines are widely used in chicken farms in China to prevent H9N2 AIV infection, including A/chicken/Guangdong/SS/94, A/chicken/Shandong/6/96, and A/chicken/Shanghai/F/98 [37], H9N2 AIV was still isolated in immunized chickens. At present, H9N2 has replaced H5N6 and H7N9 as the most popular AIV subtypes in chickens and ducks in China [30]. Since 2010, chicken-derived H9N2 viruses have accounted for the majority of H9N2 strains [19]. Our Bayesian analysis results also confirmed the above points, suggesting that chickens and ducks may play an important role in the spread of H9N2 AIVs in China. SS94 and F98 viruses mainly replicated in the respiratory tract. Oropharyngeal virus shedding lasted for 7–9 days, and cloacal virus shedding occurred occasionally [38]. In general, after H9N2 avian influenza virus infects chickens, cloacal shedding lasts for 3–5 days, while oropharyngeal shedding can last for 1–7 days [2]. Our results showed that G57 and G118 H9N2 AIVs replicated in multiple organs of chickens, but preferentially replicated in the respiratory tract. Although the viral shedding titre was not as high as that of the oropharynx, the cloacal shedding of current G57 and G118 H9N2 viruses in this study lasted for 1–7days. The extension of viral shedding time can increase the risk of virus transmission, which is worthy of our attention.

At present, H9N2 AIV has become the second most prevalent subtype causing human infections [39]. Although the H9N2 AIV has low pathogenicity to poultry, it can cause severe disease in humans. In addition to obtaining preferential binding to human-like receptors, mutations in some key amino acids are also crucial for the pathogenicity of H9N2 AIV to mammals. MDCK cells are the gold standard for influenza virus isolation, which supports the efficient replication of influenza virus and is suitable for comparing the growth kinetics of different H9N2 strains. A549 human lung epithelial cells express both avian-type (α-2,3) and human-type (α-2,6) receptors (the latter is dominant), which can evaluate the replication potential of viruses in human respiratory cells and provide insights into mammalian adaptation and cross-species transmission. D195N, D253N and I292 V mutations of PB2 protein can enhance the replication of AIV in 293 T cells or A549 cells [40–42]. The D253N and Q591 K mutants of PB2 protein of H9N2 virus have synergistic effects in enhancing polymerase activity, TNF-α production in human macrophages, replication in human bronchial epithelial cells, and pathogenicity in mice [43]. The PB2 protein contains the A588 V substitution which has been associated with enhanced polymerase activity in human cells and enhanced virulence in mice [44]. The K356R mutation of PA protein enhanced the pathogenicity of the virus to mice and the replication ability in A549 and MDCK cells [45]. In this study, CK45/20 obtained D195N, D253N, I292 V and A588 V in PB2 protein. CK1/21 obtained Q591 K mutant in PB2. Except for CK1/21, the other 7 strains obtained I292 V. Except for CKSD3/21 and CKSD2/22, the other 6 strains obtained A588 V of PB2 protein. Except for CK45/20, CKSD3/21 and CKSD2/22, the other 5 strains obtained K356R mutation of PA protein. These mutations can increase the replication of H9N2 virus in mammalian cells and enhance the pathogenicity of the virus to mammals. In vitro replication experiments showed that the current H9N2 virus could replicate efficiently in MDCK, A549 and HBE cells, and the titre of CK225/22 was higher than that of the other strains. In vivo experiments showed that the current G57 and G118 genotype H9N2 viruses replicated effectively in the turbinate and lungs of mice and caused body weight loss. In summary, our results showed that the current H9N2 virus has obtained some mutations adapted to mammals, increasing the potential threat to public health.

## Supplementary Material

Supplemental Material

Supplementary Figure 1.tif

Supplementary Figure 2.tif

Supplementary Table 1.xlsx

Supplementary Table 3.xlsx
