# Supplementary material for: Genetic evolution, phylodynamics, geographic spread of H9N2 avian influenza viruses in China from 2014 to 2025: an increasing potential zoonotic risk
Source: Emerg Microbes Infect. 2026 Aug 3;15(1):2713320. doi: 10.1080/22221751.2026.2713320 (PMC13470927; doi:10.1080/22221751.2026.2713320)
Supplement: Supplementary file 1 [file TEMI_A_2713320_SM5843.pdf]

Supplementary table 2. Viruses in this study

Virus	Genotype	Host	Time	Location	Acession number	Abbreviation
A/chicken/Qingyuan/21/2020	G118	chicken	2020	Guangdong	EPI ISL 20447203	CK21/20
A/chicken/Guangzhou/201/2020	G118	chicken	2020	Guangdong	EPI ISL 20447204	CK201/20
A/chicken/Guangzhou/45/2020	G118	chicken	2020	Guangdong	EPI ISL 20447205	CK45/20
A/chicken/Qingyuan/141/2020	G118	chicken	2020	Guangdong	EPI ISL 20447206	CK141/20
A/chicken/Kaiping/1/2021	G118	chicken	2021	Guangdong	EPI ISL 20447207	CK1/21
A/chicken/Shandong/SD3/2021	G57	chicken	2021	Shandong	EPI ISL 20447208	CKSD3/21
A/chicken/Shandong/SD2/2022	G57	chicken	2022	Shandong	EPI ISL 20447209	CKSD2/22
A/chicken/Shandong/225/2022	G118	chicken	2022	Shandong	EPI ISL 20447210	CK225/22

Supplementary table 4. HI titers of sera collected from chicken

Viruses	chicken A	chicken B	chicken C	chicken D	chicken E
CKSD3/21	1:10240	1:5120	1:10240	1:10240	1:5120
CKSD2/22	1:5120	1:5120	1:5120	1:2560	1:2560
CK/201/20	1:10240	1:10240	1:5120	1:5120	1:5120
CK225/22	1:10240	1:5120	1:10240	1:10240	1:2560

Note：The animals were euthanized at 14 days post-infection (dpi), and serum samples were collected for serological analysis. HI assays were performed used the same virus

as the inoculated strain as the antigen. HI titer was shown for individual serum sample.

Supplementary table 5. HI titers of sera collected from mice

Viruses	mouse A	mouse B	mouse C	mouse D	mouse E
CK225/22	1280	640	1280	640	320
CKSD3/21	640	1280	640	640	160
CKSD2/22	640	640	1280	1280	640

Note：The animals were euthanized at 14 days post-infection (dpi), and serum samples were collected for serological analysis. HI assays were performed used the same virus as the inoculated strain as the antigen. HI titer was shown for individual serum sample.
